# Genotype and Phenotype Heterogeneity in Neonatal Diabetes: A Single Centre Experience in Turkey

**DOI:** 10.4274/jcrpe.galenos.2020.2020.0093

**Published:** 2021-02-26

**Authors:** Yasemin Denkboy Öngen, Erdal Eren, Özgecan Demirbaş, Elif Sobu, Sian Ellard, Elisa De Franco, Ömer Tarım

**Affiliations:** 1 Bursa Uludağ University Faculty of Medicine, Department of Pediatric Endocrinology, Bursa, Turkey; 2 University of Exeter Medical School, Institute of Biomedical and Clinical Science, Exeter, United Kingdom; 3 Royal Devon and Exeter NHS Foundation Trust, Genomics Laboratory, Exeter, United Kingdom

**Keywords:** Neonatal diabetes, genetic, sulfonylurea, monogenic diabetes, potassium channel, syndromic neonatal diabetes

## Abstract

**Objective:**

Neonatal diabetes mellitus (NDM) may be transient or permanent, and the majority is caused by genetic mutations. Early diagnosis is essential to select the patients who will respond to oral treatment. In this investigation, we aimed to present the phenotype and genotype of our patients with NDM and share our experience in a single tertiary center.

**Methods:**

A total of 16 NDM patients from 12 unrelated families are included in the study. The clinical presentation, age at diagnosis, perinatal and family history, consanguinity, gender, hemoglobin A1c, C-peptide, insulin, insulin autoantibodies, genetic mutations, and response to treatment are retrospectively evaluated.

**Results:**

The median age at diagnosis of diabetes was five months (4 days-18 months) although six patients with a confirmed genetic diagnosis were diagnosed >6 months. Three patients had *KCNJ11* mutations, six had *ABCC8* mutations, three had *EIF2AK3* mutations, and one had a de *novo* INS mutation. All the permanent NDM patients with *KCNJ11* and *ABCC8* mutations were started on sulfonylurea treatment resulting in a significant increase in C-peptide level, better glycemic control, and discontinuation of insulin.

**Conclusion:**

Although NDM is defined as diabetes diagnosed during the first six months of life, and a diagnosis of type 1 diabetes is more common between the ages of 6 and 24 months, in rare cases NDM may present as late as 12 or even 24 months of age. Molecular diagnosis in NDM is important for planning treatment and predicting prognosis. Therefore, genetic testing is essential in these patients.

**What is already known on this topic?**

Neonatal diabetes mellitus is defined as diabetes diagnosed during the first six months of life. The most frequent mutations in Europe are reported to affect the ATP-dependent potassium channel genes. Genetic testing is essential for diagnosis and management.

**What this study adds?**

Neonatal diabetes mellitus can, in rare cases, arise after the age of six months. In our cohort, most of the patients had ATP-dependent potassium channel mutations similar to the literature. Genetic results in these patients lead to improved treatment with a transition to sulphonylurea therapy in those likely to benefit.

## Introduction

Diabetes presenting in the first six months of life is classified as neonatal diabetes mellitus (NDM) (1,2,3,4). Its incidence in Europe is reported to be 1:90,000 (5). NDM may be transient or permanent with about 50-60% of NDM being transient (2,3). Although most cases remit within three months after diagnosis, about 50% of the patients relapse later in life, and most frequently during adolescence (5). Insulin treatment is usually required during the first few days following initial diagnosis, but it is life-long after relapse (3,4).

The incidence of permanent NDM in the Middle East is more than in Europe at 1:21,000 (6). To date, mutations in more than 25 genes have been reported to cause NDM (7,8). The most frequent mutations in Europe are reported to affect the pancreatic ATP-dependent potassium channel genes (*KCNJ11* and *ABCC8*), and most of them are spontaneous mutations (9). Early diagnosis is essential because NDM due to these mutations is responsive to sulphonylurea (SU) treatment, and early treatment improves neurocognitive development (10,11,12,13,14,15).

In this investigation, we present the genotypic and phenotypic characteristics of patients with NDM, followed at the pediatric endocrinology clinic of Bursa Uludağ University Hospital.

## Methods

### Patients

A total of 16 NDM patients from 12 unrelated families were included in the study. Clinical data were obtained from medical records, and a consent form for genetic analysis was filled out by all parents and participants. Patients diagnosed with diabetes below the age of 12 months and/or those with infantile diabetes with syndromic features and/or those with a family history of NDM were included in the study. The clinical presentation, age at diagnosis, perinatal and family history, consanguinity, gender, glycated hemoglobin (HbA1c), C-peptide, insulin, and insulin autoantibodies, genetic mutations, and response to treatment were retrospectively evaluated. Informed consent for genetic testing was obtained from the parents. The study was approved by the Ethical Committee of Bursa Uludağ University (approval number: 2020-8/23).

### Laboratory Analysis

Serum glucose was analyzed by spectrophotometric methods (C16000 Architect System, Abbott, USA). C-peptide and insulin were assessed with chemiluminescent microparticle immunoassay (i2000 Architect System, Abbott, USA). HbA1c was measured by high-pressure liquid chromatography (Hb9210 Trinity Biotech Premier, USA). Glutamic acid decarboxylase antibody (GAD-65) and anti-insulin antibody were performed by enzyme immunoassay (DiaSarin ETI-MAX 3000, Italy). Pancreatic islet cell antibody was studied by indirect fluorescent antibody method.

### Genetic Analysis

Analysis of all coding regions and exon/intron boundaries of the *KCNJ11, ABCC8, INS* and *EIF2AK3* genes was performed by Sanger sequencing. Genetic testing for all known genetic causes of NDM for eight of the patients was performed by the Exeter genomic laboratory, as previously described (16). The clinical significance of the variant was assessed using the Association for Clinical Genomic Science Best Practice Guidelines for Variant Classification 2019 (17).

### Statistical Analysis

Descriptive analysis was performed using SPSS, version 21.0 (IBM Inc., Armonk, NY, USA). Data were expressed as median (minimum-maximum range) or mean±standard deviation (range).

## Results

The median age at diagnosis of diabetes for the whole cohort (n=16) was five months (4 days to18 months), and the female to male ratio was 1.3:1. The mean glucose level at diagnosis was 475±137 mg/dL. Nine patients presented with diabetic ketoacidosis (DKA), two patients with ketosis, and four with hyperglycemia. One patient was diagnosed elsewhere, and the initial presentation was not known (patient 12.15). The median HbA1c at the time of diagnosis was 10.2% (5.8-17.1%), and the median C-peptide was 0.085 ng/mL (0.01-1.22 ng/mL) (reference range 0.78-5.19 ng/mL). Eleven patients were born full-term, three of them with low birth weight (<2,500 g), and five with a birth weight of 2,500-3,500 g. The gestational age and birth weight of four patients were not available. Multiple insulin regimens such as intermediate-acting (NPH), rapid-acting (insulin lispro) and short-acting insulin (regular), were started in 15/16 of the patients. Only one patient was treated with an insulin pump. Pancreatic imaging (sonographic examination) was performed in all of the patients, and none of them showed pancreatic abnormality. A genetic test was performed in 15 patients (Table 1).

A mutation in a gene known to cause NDM was identified in thirteen (86.7%) patients, but for two patients testing for all the known NDM genes did not detect a likely causative mutation. These patients without a mutation identified were diagnosed at the age of seven months and four days, respectively, and were both positive for anti-GAD antibodies (concentrations were 26.5 and 53.95 IU/mL, normal level <5 IU/mL) (patients number 7.9 and 9.11 in Table 1). Although anti-GAD antibodies were positive, anti-insulin antibodies were in the normal range (concentrations were 0.2 and 2.5 IU/mL, normal level 0-10 IU/mL). Their birth weights were 3,700 g and 2,300 g, and they were not significantly different from the rest of the cohort.

Three unrelated patients had the *KCNJ11* mutations, six (including three from the same kinship) had *ABCC8 *mutations, three had *EIF2AK3* mutations, and one had a *de novo INS *mutation.

### Patients with ATP-Dependent Potassium Channel Mutations

Patient 6.6 was diagnosed with ketosis at 18 months of age and was on insulin treatment until she was 17 years old when she was found to be homozygous for a previously reported *ABCC8* mutation classified as pathogenic (p.Glu382Lys) and switched to SU treatment. She had two cousins with diabetes on insulin treatment at 18 and 24 years of age who were also diagnosed during infancy (patients number 6.7 and 6.8). These patients were also found to be homozygous for the *ABCC8* pathogenic variant and switched to SU. These three patients all responded well to oral treatment, and insulin was successfully discontinued.

One patient, diagnosed at twelve days of age with a previously reported *ABCC8* heterozygous mutation (p.Arg1183Gln), was off-treatment at four months of age, confirming transient NDM (patient 8.10).

Two sisters, diagnosed with NDM at six and eight months of age, were homozygous for the p.Trp231Leu mutation in the *ABCC8* gene (patients 10.12 and 10.13). Although, this variant was not previously reported in the literature and initially classified as a variant of uncertain significance, a trial switch from insulin treatment to SU was successful and the variant could therefore be re-classified as likely pathogenic.

Three unrelated patients were found to be heterozygous for the previously reported pathogenic *KCNJ11* p.Val59Met mutation. This variant has been previously reported in patients with iDEND (18,19). However, none of our patients was reported to have neurological features at the ages of seven, six and a half and six years.

All the permanent NDM patients with *KCNJ11* and *ABCC8* mutations were successfully transferred to SU treatment, resulting in a significant increase in C-peptide level after three months, better glycemic regulation, and discontinuation of insulin (Table 2). SU was started at a dose of 0.2 mg/kg/day, twice a day. Later, doses were adjusted with blood glucose levels. The doses of SU were in the range 0.2-1.2 mg/kg/day. Only one patient required a single dose of long-acting insulin four years after the diagnosis (patient 2.2).

### Patients with Mutations in Other Genes

 One patient with a novel heterozygous *de novo* mutation in the *INS* gene (p.Cys95Trp) was diagnosed at the age of four months. He remains insulin-treated (patient 4.4). One patient diagnosed at 15 months of age developed elevated levels of AST and ALT after one year, anemia, and leukopenia later during follow-up (patient 11.14). He also had congenital stenosis of the aorta and skeletal dysplasia, which became evident after infancy. Anti-GAD was negative, and Wolcott Rallison syndrome was confirmed by the detection of two homozygous *EIF2AK3* mutations. He is still on insulin and supportive therapy (for orthopedic complications and autoimmune hepatitis) at the age of 14.5 years. Similarly, another unrelated patient diagnosed at 15 months of age showed elevated transaminase levels, persistent hyperkalemia, thrombocytopenia, and skeletal dysplasia after two years and was also found to be homozygous for an *EIF2AK3* mutation (patient 12.15). His sister, diagnosed with NDM at four months of age, was homozygous for the same mutation (patient 12.16). Both patients are still on insulin and supportive treatment (for orthopedic and renal complications) at the age of 15.5 and 4.5 years.

## Discussion

Although NDM is defined as diabetes diagnosed during the first six months of life, recent research has shown that, rarely, it may present as late as 12 or even 24 months of age (1,2,3,4) although between the ages of six and 24 months a diagnosis of type 1 diabetes is much more common. The median age of diagnosis in our study was five months (four days-18 months). The most striking finding in this investigation was the presentation of diabetes in a patient with genetically confirmed NDM at 18 months of age. This patient and his two cousins were found to have a homozygous pathogenic *ABCC8* mutation, and after many years on insulin treatment, they were successfully switched to SU therapy. NDM genes must therefore be considered when carefully collected family history suggests a possible genetic cause. 

There was no statistical difference in terms of gender in our patients. Iafusco et al (20) similarly reported no gender difference in their cohort.

In a study reported by Russo et al (21), 75% of the patients with NDM diagnosed during the first six months of life had a mutation in *KCJN11*, *ABCC8*, or *INS* gene. This ratio dropped to 12% in patients diagnosed between 7-12 months. The same study also reported that the patients diagnosed with permanent NDM before six months of age but without mutations in *KCJN11*, *ABCC8*, or *INS* had higher birth weight than those with the mutations. In our smaller cohort, we did not observe a similar difference between patients with and without a causative mutation. Similarly to Besser et al (22), more than 50% of our patients with NDM had low birth weight despite term delivery, likely due to *in utero* hypoinsulinemia. Letourneau et al (23) reported that 66.2% of the patients with monogenic diabetes presented with DKA, similar to our patients, with 60% having DKA at the time of diagnosis. 

Previous reports have suggested that autoantibodies are usually negative in NDM patients diagnosed before six months of age, except for maternal autoantibodies, which may have crossed the placenta (24) and patients with monogenic autoimmunity such as IPEX syndrome (25). GAD-65 were positive, but anti-insulin antibodies were negative, in two of our patients diagnosed at seven months and four days (the patients’ number 7.9 and 9.11). These patients did not have mutations in the known NDM genes (including monogenic autoimmunity genes such as *FOXP3, IL2RA, *and* LRBA), *however more causal genes remain to be discovered and a monogenic etiology is therefore possible. Whilst the antibody positivity in the patient diagnosed at seven months suggests a diagnosis of type 1 diabetes is likely, further investigations are needed to define the genetic etiology of the patient diagnosed at four days, since antibody positivity is common in patients with NDM caused by monogenic autoimmunity (26).

Molecular diagnosis in NDM is important for planning treatment and predicting prognosis. Therefore, genetic testing is essential in these patients. Carmody et al (13) have discussed the pros and cons of trying SU treatment awaiting the results of genetic tests. The advantages are a neurologic improvement, shorter hospital stay, lower cost, easier than insulin injections, and safety. On the other hand, increased expectance and disappointment of the family in case of treatment failure, risk of hypoglycemia in transient NDM, unknown long-term risks, and lack of FDA approval for SU in infants are the disadvantages. One of our patients was started on SU and responded well before the genetic test result was obtained, and insulin was successfully discontinued (patient 5.5). After receiving the test results, all of the patients were switched to SU, and better glycemic control was achieved along with significant elevation in C-peptide. Other family members with diabetes were also tested and switched to SU, which markedly improved their quality of life. Bowman et al (15) published a cohort of 90 patients with *KCNJ11* mutations causing permanent NDM and followed for ten years. SU response was excellent in 93%, and neurologic development was improved by 47%. Similarly, we observed an excellent response to SU in 7/8 (87.5%) patients with mutations affecting the pancreatic potassium channel. Only one patient required the addition of long-acting insulin to the treatment. 

Despite the importance of genetic diagnosis, it may not be possible in all patients as some etiological genes still remain to be discovered. De Franco et al (16) reported that a genetic mutation was detected in 82% of patients in an international cohort of 1200 probands. Similarly, we found a causative mutation in 87% of our patients.

The most common syndromic form of NDM in countries with high consanguinity rate is Wolcott Rallison syndrome (16). We had three patients with this syndrome, including two siblings born to consanguineous parents. All three patients had hepatic dysfunction and skeletal dysplasia, which are known features of the syndrome (27). They are on insulin treatment, and their diabetes is well controlled. Demirbilek et al (28) investigated the genetic profile of the patients with NDM in Southeastern Turkey and found that mutations in potassium channel were less common in consanguineous families, while syndromic diabetes was more common. In our cohort, potassium channel mutations were more common, similarly to what is reported from Western countries.

### Study Limitations

There were some difficulties in obtaining complete data because of the retrospective nature of the study. The age range of the patients was wide, and some patients had antibody positivity, which rendered patient selection for the study difficult. The relatively small number of patients in this cohort is limited, and further, ideally prospective, research with larger numbers of patients is warranted.

## Conclusion

The recognition of NDM has increased with the identification of new genetic causes and the wider availability of genetic testing. Early diagnosis is essential to identify the patients who may respond to SU treatment. NDM has been defined as diabetes diagnosed during the first six months of life, but it is now increasingly recognized that the presentation of NDM may be delayed. In rare cases, it may present as late as 12 or even 24 months of age. Therefore very careful investigation of family history is essential. However, most patients still present before six months of age, and rapid genetic diagnosis must be obtained to plan the treatment. Syndromic diabetes must be considered in those with additional findings.

## Figures and Tables

**Table 1:**
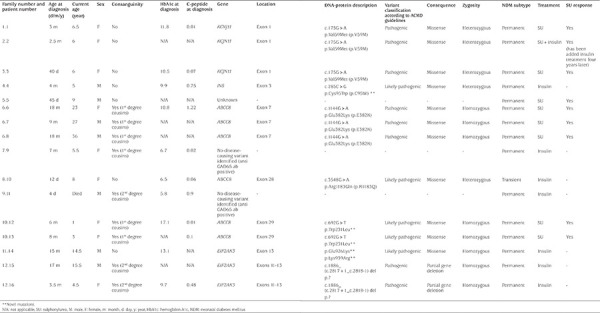
The age of diagnosis, genetic analysis, and treatment response of neonatal diabetes mellitus patients

**Table 2:**
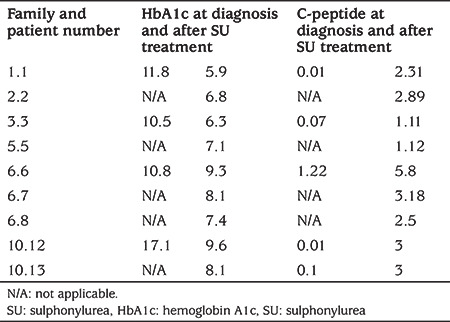
Values of C-peptide and hemoglobin A1c levels of neonatal diabetes mellitus patients with ATPdependent potassium channel mutations before and after sulphonylurea treatment
